# Construction of Metal‐Organic Frameworks (MOFs)–Based Membranes and Their Ion Transport Applications

**DOI:** 10.1002/smsc.202000035

**Published:** 2021-01-18

**Authors:** Lulu Fu, Zhao Yang, Yuting Wang, Ruirui Li, Jin Zhai

**Affiliations:** ^1^ Key Laboratory of Bio-Inspired Smart Interfacial Science and Technology of Ministry of Education Beijing Advanced Innovation Center for Biomedical Engineering School of Chemistry Beihang University Beijing 100191 P. R. China; ^2^ School of Energy and Power Engineering Beihang University Beijing 100191 P. R. China

**Keywords:** biosensing, energy conversion, ion transport, metal-organic frameworks, separation

## Abstract

Biological ion channels with delicate ion transport functions exist widely in living organisms. Bioinspired solid‐state channels have emerged as promising platforms to mimic the working mechanism of natural channels. These channels help in understanding the mechanism of ion transport in biosystems and pave the way for applications in several interesting areas. Metal‐organic frameworks (MOFs), constructed by connecting metal ions and organic ligands, exhibit high porosity and unique pore structures. They also possess tunable pore sizes and are versatile in their compositions. And there are rich molecule/ion‐specific functional groups in the frameworks, which can intelligently modulate ion transport. Thus, MOFs are promising candidates that mimic biological ion channels. Herein, the recent progress in the design and construction of artificial solid‐state MOF‐based channels is focused on. The excellent ion transport properties of MOF‐based membranes are also summarized, including ionic selectivity, ionic rectification, and ionic gating. Following this, four emerging ion transport applications, namely, energy conversion, ion/molecule separation, biosensing, and water desalination, are highlighted. Finally, an outlook on future developments and challenges in this exciting research area is presented.

## Graphical abstract

Artificial metal‐organic framework (MOF)‐based channels exhibit similar ion transport properties to biological ion channels and can be used as promising platforms for highly selective and efficient ion transportation studies. Herein, recent research progress in the fabrication of artificial MOF‐based channels is presented and emerging ion transport applications, including energy conversion, separation, biosensing, and water desalination, are highlighted.

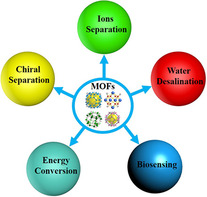

## 1 Introduction

The natural protein ionic channels in biological membranes exhibit extraordinary selectivity and efficient ions transportation ability due to the highly specific control of ionic interactions in the confined pores.^[^ ^1^, ^2^, ^3^, ^4^ ^]^ Biological membranes provide inspiration for designing artificial solid‐state membranes with biomimetic channel structures that can potentially exhibit specific selectivity and ultrahigh permeability for small molecules and ions similar to the biological membranes.^[^ ^5^, ^6^, ^7^, ^8^ ^]^ To date, numerous artificial solid‐state membranes, such as inorganic‐ and organic‐based hybrid nanochannel membranes, have been synthesized for various applications.^[^ ^9^, ^10^, ^11^, ^12^, ^13^, ^14^, ^15^, ^16^ ^]^ However, they suffer from plentiful defects, including intricate fabrication steps and limitative material selections. ^[^ ^17^, ^18^, ^19^ ^]^ Therefore, it is important to explore novel functional materials with excellent characteristics in an easily available and versatile way.

**Figure 1:**
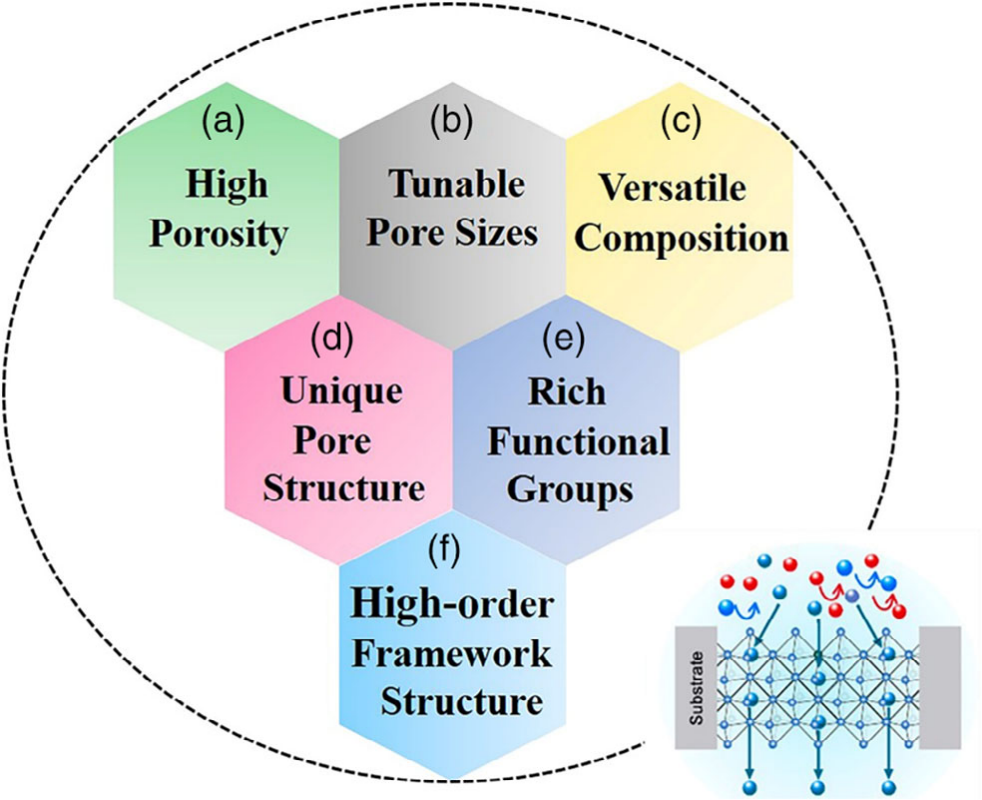
a–f) Merits of MOFs for ions transport applications.

Particularly, metal‐organic frameworks (MOFs) are considered promising candidates for the development of advanced materials for highly selective and efficient ion transportation studies.^[^ ^20^, ^21^, ^22^ ^]^ MOFs are composed of metal ions and organic linkers. They possess inherently high porosity and unique pore structures. They exhibit a narrow size distribution and contain molecule/ion‐specific functional groups (**Figure** **1**).^[^ ^23^, ^24^, ^25^ ^]^ Thus, they can potentially provide an excellent platform to realize functions that are analogous to the functions of biological membranes. It has been reported that the selective transport of ions through MOFs depends on their accurately defined pore sizes.^[^ ^26^, ^27^ ^]^ In general, the ion transport mechanisms of MOFs can occur in two ways. First, MOF membranes assist the size‐selective separation of molecules and ions when the pore sizes of the MOF membranes are comparable to the kinetic diameters of the species being separated. Second, the molecule/ion‐specific functional groups present in the MOF frameworks can further improve the membrane selectivity.

In such artificial systems, ion transport can be regulated to achieve high‐level intelligence and powerful functionalities exhibiting great potential in practical applications.^[^ ^28^, ^29^ ^]^

In this review, we present the advances of engineering MOF‐based membranes for ion transport studies. To demonstrate this, the review is divided into four sections. In the first section, we briefly introduce artificial solid‐state MOF‐based membranes and the generic construction strategy. In the second section, we introduce in detail the excellent ionic transport properties of artificial solid‐state MOF‐based membranes for ionic transport. Following this, we present four emerging ions transport applications of the MOF‐based membrane, including energy conversion, separation, biosensing, and water desalination. Finally, a perspective is given on future challenges and opportunities in the development of artificial solid‐state MOF‐based membranes.

## 2 Construction of MOF‐Based Membranes

**Figure 2:**
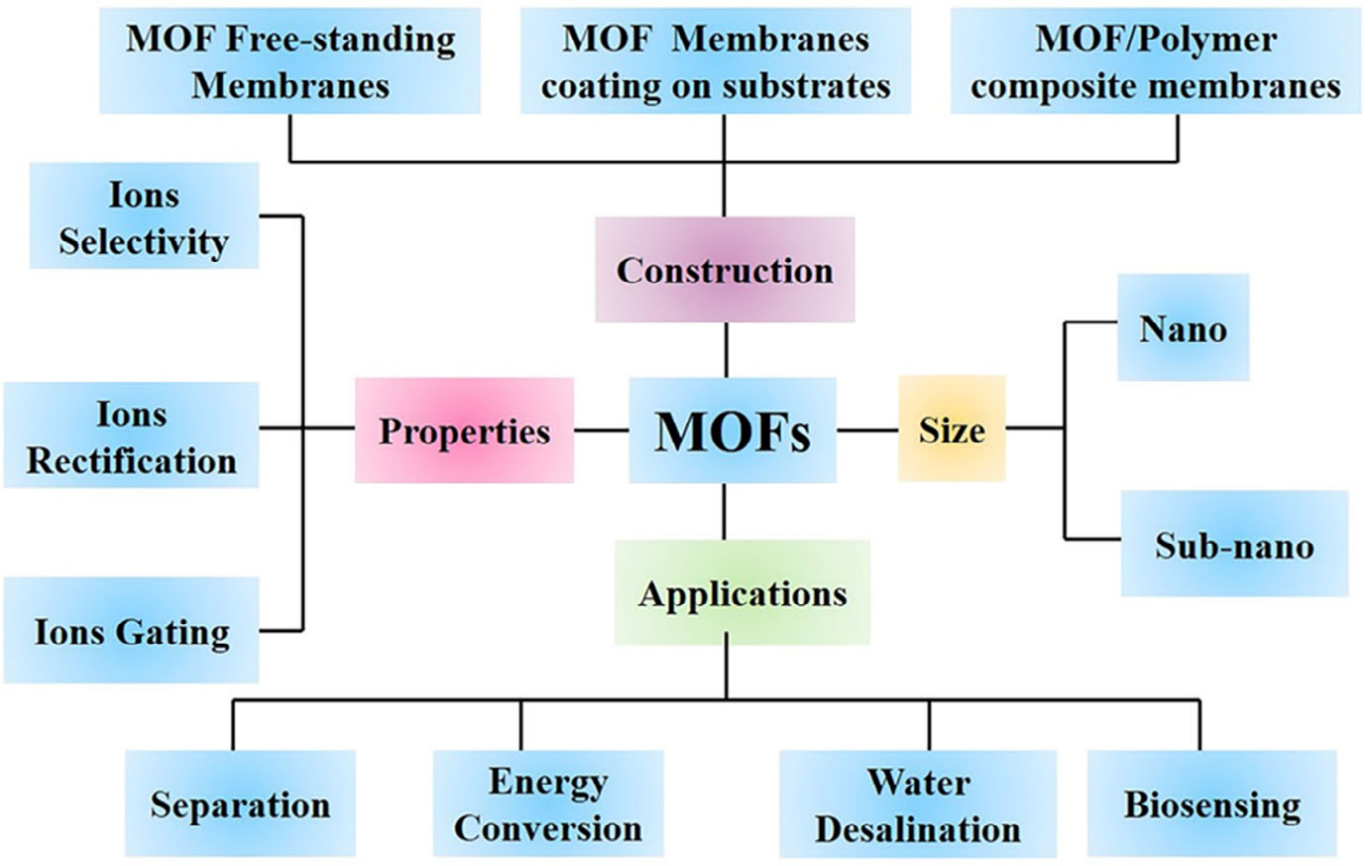
Generic strategy for constructing MOF‐based membranes.

MOFs have been rapidly emerging as a very powerful platform for constructing multifunctional materials.^[^ ^30^, ^31^ ^]^ These frameworks with tunable pore sizes and functional surfaces have emerged as promising materials for confined ion transport studies.^[^ ^32^ ^]^ In the past few decades, several studies have been conducted to synthesize high‐quality MOF membranes using various methods (such as solvothermal synthesis, hot pressing, secondary growth, and contra‐diffusion synthesis and electrochemical synthesis).^[^ ^33^, ^34^, ^35^, ^36^, ^37^ ^]^ In general, the design strategy for constructing MOF‐based membranes is divided into three categories: free‐standing MOF membranes, MOF/polymer mixed matrix membranes (MMMs), and MOF membrane coating on substrates (**Figure** **2**).

The fabricated MOF‐based membrane systems can exhibit excellent ionic transport properties such as ion selectivity, ion gating, and ion rectification, similarly to biological counterparts in nature.^[^ ^38^, ^39^ ^]^ Investigations of the pores formed in MOF‐based membranes have revealed that their sizes range from the subnanometer to nanometer scale, and the pores serve as selective channels for rapid permeation of particles. Notably, the artificial solid‐state MOF‐based membranes exhibit potential ion transport applications, including energy conversion, ion/molecular separation, biosensing, water desalination, etc.^[^ ^40^, ^41^, ^42^, ^43^, ^44^, ^45^ ^]^

**Figure 3:**
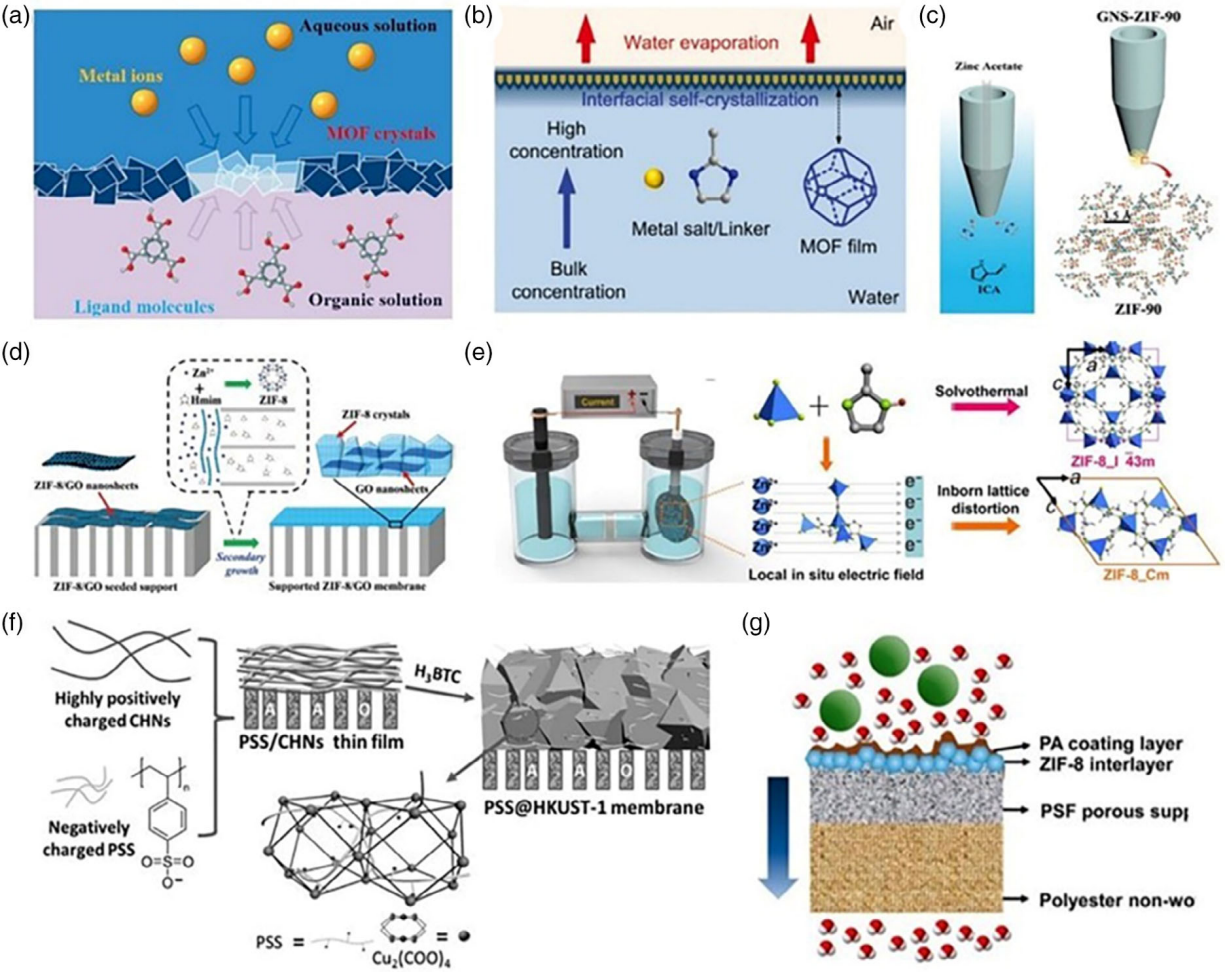
Recent advances made in the development of MOF‐based membranes. a) Schematic illustration of the liquid–liquid interfacial fabrication method of an MOF membrane using a biphasic mixture consisting of an aqueous metal ion containing solution and an organic ligand solution. b) Schematic representation of the air–liquid interfacial fabrication ZIF‐8 crystallization. c) Schematic illustration of the procedure followed for the synthesis of ZIF‐90 at the tip of the glass nanopipette. d) Schematic diagram of the process followed for the synthesis of ultrathin ZIF‐8/graphene oxide (GO) membrane: coating of ZIF‐8/GO nanosheets onto AAO supports and subsequent secondary growth into a continuous membrane through a contra‐diffusion method. e) Schematic diagram showing the growth of ZIF‐8 membranes on AAO porous substrates via electrochemical method. f) Schematic illustration of the fabrication steps of polystyrene sulfonate (PSS)@1‐(2‐methyl‐4‐(2‐oxopyrrolidin‐1‐yl)phenyl)‐3‐morpholino‐5,6‐dihydropyridin‐2(1H)‐one (HKUST‐1) composite membranes. g) Schematic illustration of the fabrication steps of MOF/polyethylene terephthalate (PET) hybrid channels via the contra‐diffusion growth method. (a) Reproduced with permission.^[^ ^46^ ^]^ Copyright 2011, Springer Nature. (b) Reproduced with permission.^[^ ^48^ ^]^ Copyright 2019, Royal Society of Chemistry. (c) Reproduced with permission.^[^ ^62^ ^]^ Copyright 2017, Wiley‐VCH. (d) Reproduced with permission.^[^ ^63^ ^]^ Copyright 2016, Wiley‐VCH. (e) Reproduced with permission.^[^ ^64^ ^]^ Copyright 2018, AAAS. (f) Reproduced with permission.^[^ ^68^ ^]^ Copyright 2016, Wiley‐VCH. (g) Reproduced with permission.^[^ ^69^ ^]^ Copyright 2015, American Chemical Society.

The liquid–liquid interface synthesis method is most commonly used for fabricating free‐standing MOF membranes at the interface between two liquid phases. One of the phases contains metal ions and the other contains organic linkers.^[^ ^46^, ^47^ ^]^ For example, Ameloot et al. synthesized a hollow capsule‐shaped free‐standing [Cu_3_(BTC)_2_] MOF membrane using the liquid–liquid interface synthesis method. Two immiscible solvents were used for the process. Each of the solvent phases contained Cu (II) ions and 1,3.5‐benzenetricarboxylate (BTC) organic ligands (**Figure** **3**a).^[^ ^46^ ^]^ The differences in the solubilities of the organic and inorganic precursors assist the self‐completing interfacial formation of an MOF layer in a biphasic synthesis mixture. Such an MOF membrane exhibited size‐selective permeability, which can be potentially exploited for the development of selective microreactors. Similar to the liquid–liquid interface synthetic approach, the air–liquid interface synthesis method is also used for preparing MOF films.^[^ ^48^, ^49^, ^50^ ^]^ Recently, Li and co‐workers reported the fabrication of zeolitic imidazolate framework (ZIF)‐8 membranes at the air–water interface through the process of self‐crystallization (Figure 3b).^[^ ^48^ ^]^ This work provides valuable insights into the development of advanced membranes possessing unique features that can be exploited to extend the application of MOFs in various fields such as membrane separation and energy conversion. The fabricated free‐standing MOF membranes using interfacial synthesis maintain a high level of permanent porosity as well as the intrinsic functions and properties of MOFs, which can be fully utilized without any redundant modifications or other supports. Nevertheless, in many cases, pure free‐standing MOFs cannot meet the requirements of practical applications due to their intrinsic limitations such as poor mechanical strength and flexibility as well as limited functionality. Advanced technologies for preparing high‐quality, pure free‐standing MOF membranes need to be further developed to meet the high requirements of durability and stability in practical applications.

Recent efforts have demonstrated that the combination of pure MOFs with substrate materials to construct MOF‐based composite membranes can overcome the disadvantages of pure MOFs while retaining the original merits of MOFs.^[^ ^51^, ^52^, ^53^ ^]^ The porous substrates exhibit minimal permeation resistance and provide mechanical support to the MOF layer. To date, substrates such as inorganic oxide solids and flexible organic membranes have been successfully used for fabricating MOF layers.^[^ ^54^, ^55^, ^56^, ^57^, ^58^ ^]^ Moreover, numerous organic functional groups are also used for modifying the substrates, such as carboxyl, hydroxy, and amino groups, which can bind to metal ions or organic linkers and thus nucleate the growth of MOF thin membranes. Plentiful synthetic strategies have been developed for fabricating MOF films on porous substrates.^[^ ^59^, ^60^, ^61^ ^]^ In addition, in situ synthesis methods have also been used for fabricating MOF‐based membranes. Wang and co‐workers used the in situ growth method to fabricate a crack‐free subnanometer ZIF‐90 composite at the tip of glass nanopipettes to study ion transport (Figure 3c).^[^ ^62^ ^]^ This work offers a general and convenient way to study the migration behavior of ions through the subnanometer channels, demonstrating its promising application in investigating the transport of molecules/ions, gas, and the catalytic process in confined spaces. The contra‐diffusion synthesis method has also been used for preparing MOF‐based composite membranes. For example, Wang's group fabricated ultrathin and defect‐free ZIF‐8‐based composite membranes, utilizing 2D ZIF‐8/GO nanosheets as the seeding layer on an anodic aluminum oxide (AAO) support and subsequent secondary growth via contra‐diffusion synthesis (Figure 3d).^[^ ^63^ ^]^ The seeding layer was prepared through 2D hybrid ZIF‐8/GO nanosheets coated on the surface of the porous AAO support by spin‐coating method. Subsequently, a ZIF‐8/GO membrane with a thickness of ≈100 nm was obtained via 3 h secondary growth. The ZIF‐8/GO nanosheet acts as a barrier between different solutions including metal ions and ligands, which can effectively avoid crystal overgrowth and defects during the contra‐diffusion process, contributing to a defect‐free and ultrathin membrane. The GO nanosheets offer a confined space, facilitating crystal intergrowth, which significantly enhances the mechanical toughness of the ZIF‐8 layer. Electrochemical fabrication methods can also be used for fabricating supported MOF membranes. Most recently, Caro and co‐workers reported a fast current‐driven synthesis technique for the rapid fabrication of ZIF‐8 membranes. They produced highly intergrown and ultrathin ZIF‐8 membranes on AAO porous substrates (Figure 3e).^[^ ^64^ ^]^ This synthesis method is not only convenient, mild, and straightforward, but also a general strategy for the large‐scale production of high‐quality MOF membranes for applications ranging from separation to energy conversion.

Because pure MOF crystalline membranes lack flexibility and are difficult to process, the combination with polymers can be a good choice. The MOF/polymer composite membrane is composed of porous MOFs dispersed in a polymer matrix. The advantageous properties of both the components get incorporated into the composite membrane, which is a promising material for application in various fields.^[^ ^65^, ^66^, ^67^ ^]^ Chen and co‐workers constructed intergrown and continuous PSS‐threaded HKUST‐1 membranes following the solid confinement conversion process, which was used to separate lithium ions from binary alkali metal ion mixtures **(**Figure 3f).^[^ ^68^ ^]^ The incorporation of linear PSS enhanced the water stability of HKUST‐1 membranes and promoted the generation of 3D networks of sulfonic groups for ion transport. Recently, Lei's group reported a novel layer‐by‐layer (LBL) fabrication method to prepare a polyamide (PA)/ZIF‐8 nanocomposite membrane by growing an interlayer of ZIF‐8 nanoparticles on an ultrathin PA layer through interfacial polymerization (Figure 3g).^[^ ^69^ ^]^ This work opens an interesting pathway for the synthesis of polymer nanocomposite membranes that exhibit high performance and good selectivity.

## 3 Properties

Biological ion channels exhibit three basic ion transport characteristics, namely, ion selectivity, ion rectification, and ion gating. The proper implementation of these functions is essential for maintaining an organism's physiological activities. MOFs exhibiting high porosity, unique channel structure, tunable pore sizes, and various functional sites have attracted attention as ion transport materials that are also capable of achieving these delicate transport functions. In this section, we summarize the excellent ion transport properties of the artificial solid‐state MOF membranes.

### 3.1 Ion Selectivity

Ion selectivity refers to the ability of an ion channel to sense and detect specific types of ions or molecules. The selectivity depends on the specific binding sites asymmetrically distributed along the inner channel walls.^[^ ^70^ ^]^ Inspired by the selectivity of the biological ion channel protein, artificial smart channels that can selectively transport specific types of ions/molecules have been developed.^[^ ^71^, ^72^ ^]^

**Figure 4:**
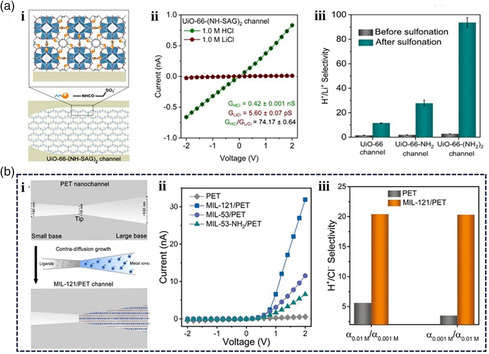
a) i. Schematic illustration of fabricating subnanometer UiO‐66–(NH‐SAG)_2_ channels. ii. *I*–*V* curves plotted for a UiO‐66–(NH‐SAG)_2_ channel in 1.0 m HCl and LiCl solution, respectively. iii. Before and after sulfonation H^+^/Li^+^ selectivity of UiO‐66, UiO‐66–NH_2_, UiO‐66–(NH_2_)_2_ channels under 0.1 m electrolyte solution. b) i. Schematic illustration of fabrication steps of MOF/PET hybrid channels. ii. *I*–*V* curves plotted for a PET nanochannel, and various MOF/PET hybrid channels measured in 0.01 m HCl solution. iii. The H^+^/Cl^−^ selectivity of PET and Materials of Institute Lavoisier (MIL)‐121/PET channel measured in 0.01 α_0.01_ M/α_0.001_ M, and α_0.001_ M/α_0.01_ M conditions. (a) Reproduced with permission.^[^ ^38^ ^]^ Copyright 2020, American Chemical Society. (b) Reproduced with permission.^[^ ^73^ ^]^ Copyright 2020, Wiley‐VCH.

Inspired by the biological proton channels, Li and co‐workers have recently designed artificial proton channels with biological‐level selectivity based on sulfonated subnanometer MOF channels.^[^ ^38^ ^]^ The subnanometer MOF channels were fabricated by assembling postsulfonated UiO‐66‐derivative MOFs into bullet‐shaped single PET nanochannel membranes (**Figure** **4**a(i)). The sulfonated UiO‐66 MOF‐based channels exhibiting angstrom‐sized windows for ion sieving and nanoconfined sulfonic acid groups for proton hopping exhibited the highest proton selectivity over other monovalent cations such as Li^+^, Na^+^, and K^+^. The UiO‐66–[NH‐sulfonic acid groups (SAG)]_2_ channel exhibited an ideal H^+^/Li^+^ selectivity up to ≈80 (Figure 4a(ii)). The ultrahigh proton selectivity can be attributed to the tethered sulfonic acid groups present in the nanoconfined window cavity of the MOF channels, serving as proton‐hopping sites and forming hydrogen‐bonded networks with water molecules inside the channel, which promote fast proton transport through the MOF channels and simultaneously exclude other cations. Furthermore, the H^+^/Li^+^ selectivity of the sulfonated UiO‐66 channels increased with a decrease in the MOF channel sizes, which was attributed to the increasing number of tethered functional sites (Figure 4a(iii)). This work provides a platform for the development of angstrom‐sized functional MOF channels exhibiting selective ion conduction and efficient ion separation.

In addition, Wang and co‐workers reported MOF/polymer heterostructured nanochannels with nano‐to‐subnano asymmetric architectures via a contra‐diffusion growth method, which realized biomimetic unidirectional, fast, and selective proton conduction properties (Figure 4b(i)).^[^ ^73^ ^]^ Notably, after filling the MOFs on one side of the PET nanochannel, the I–V curve of the MIL‐121/PET channel became highly rectified, exhibiting unidirectional proton transport (Figure 4b(ii)), with a rectification ratio of ≈500. The MIL‐121/PET heterostructured channels exhibited good proton selectivity over other cations (Figure 4b(iii)). The theoretical simulations revealed that the preferential and fast proton conduction could be attributed to the extremely low energy barriers for proton transport. This work demonstrated a novel method to regulate ion selectivity and permeability in artificial ion channels.

### 3.2 Ion Rectification

Ion rectification is also an essential characteristic of the biological ion channel. Ion rectification generally refers to the ionic current being preferentially directional ionic transport, acting as an ion diode.^[^ ^74^ ^]^ Ion rectification can be achieved by introducing asymmetric geometry and surface charge distribution.

**Figure 5:**
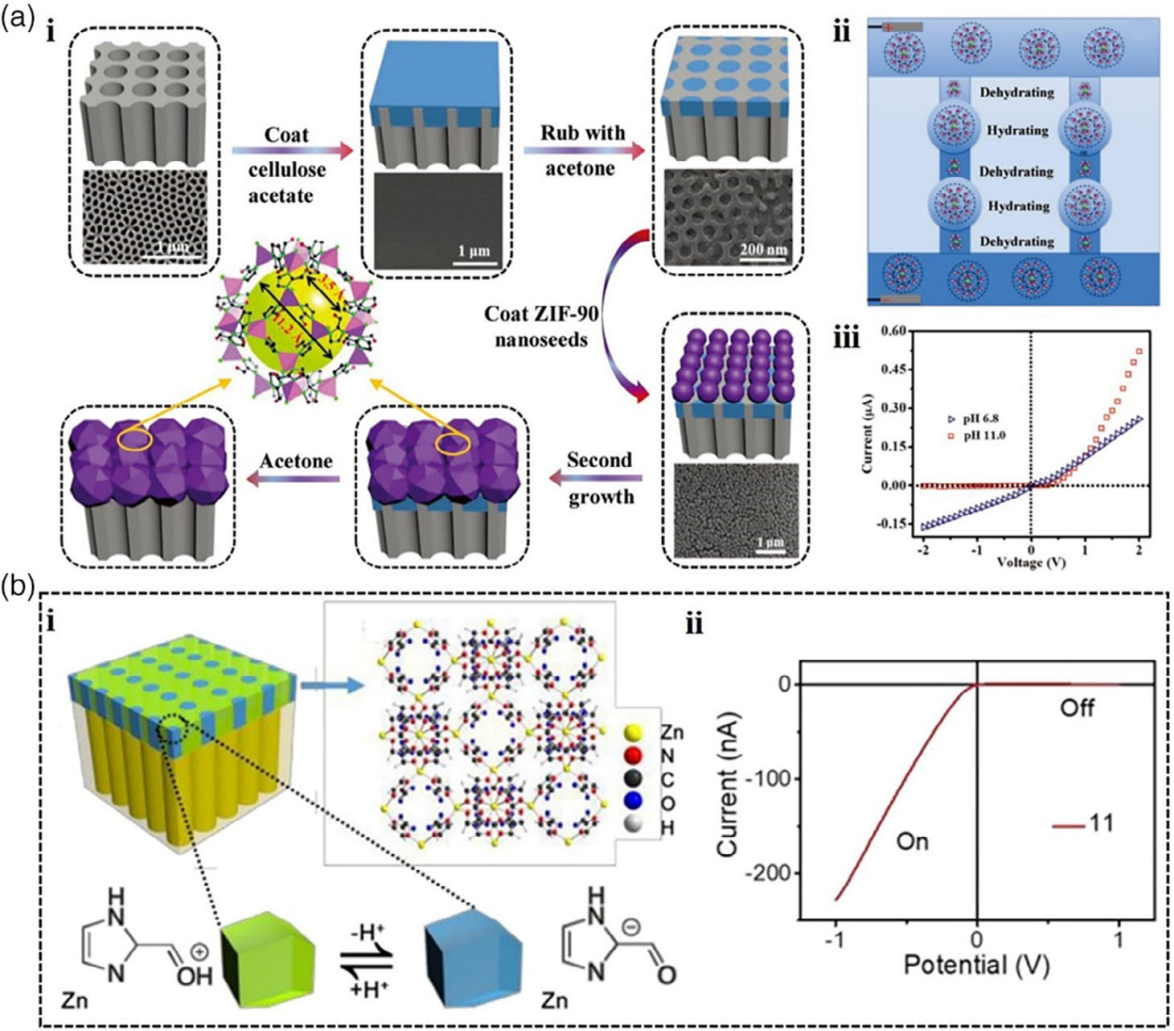
a) i. Schematic illustration of the fabrication steps of the asymmetrical membrane with angstrom‐sized pores. ii. Schematic representation of the ion transport through the ZIF‐90 pores. iii. *I*–*V* curves plotted for the asymmetrical membrane in 10 mm KCl electrolyte at different pH values. b) i. Schematic diagram of the asymmetric MOF/PAA hybrid channels. ii. *I*–*V* curves and ion rectification ratio of MOF/PAA hybrid at different pH (from 3 to 11) at 1 mm KCl electrolyte. (a) Reproduced with permission.^[^ ^75^ ^]^ Copyright 2019, Wiley‐VCH. (b) Reproduced with permission.^[^ ^76^ ^]^ Copyright 2020, Royal Society of Chemistry.

MOFs with unique structure and angstrom‐sized aperture distributions make ion channels a perfect confined space for ion transport, which exhibit distinctive ion transport behavior. When the sizes of the pores decrease to the angstrom scale, the quantum‐confined effect and ionic coulomb blockade effect can be observed. For example, inspired by the confinement effect in asymmetrical biological ion channels, our group designed an asymmetrical membrane by growing the ZIF‐90 membrane on the porous AAO film for ion separation (**Figure** **5**a(i)).^[^ ^75^ ^]^ Particularly, the ion transport through the pores of ZIF‐90 involves multiple dehydration–hydration–dehydration processes in the form of a single ionic chain. The phenomenon can be attributed to the confinement effect of angstrom‐sized aperture of ZIF‐90 (Figure 5a(ii)). Furthermore, a high rectification ratio of 237 was obtained in 10 mm KCl electrolyte with pH of 11 (Figure 5a iii). In addition, the current turning point shifted to +0.19 V, which is obviously different from the ion transport behavior in nanosized asymmetrical channels where the current turning point always appears at 0 V. The reason is that the ions that transport across the ZIF‐90 composite membrane have to overcome the energy barrier. These unique ion transport characteristics impart MOF‐based membranes with remarkable alkali metal ion separation performance. This study not only helps to understand the mechanism of ion transport through angstrom‐sized pores, but also provides an excellent platform for ion separation.

Recently, Liu and co‐workers reported a facile strategy to construct an MOF/porous anodic alumina (PAA) hybrid via in situ synthesis of a zeolitic imidazole framework (ZIF‐90) on PAA nanochannels with angstrom‐sized pores (Figure 5b(i)).^[^ ^76^ ^]^ Notably, the ions passing through the ZIF‐90 layer undergo multiple dehydration–hydration processes, because the dimensions of the hydrated diameters of K^+^ (6.62 Å) and Cl^−^ (6.64 Å) are larger than the angstrom‐sized window of ZIF‐90 (3.5 Å), whereas the dimensions of the dehydrated diameter of K^+^ (2.66 Å) and Cl^−^ (3.62 Å) are similar to the aperture of ZIF‐90. Thus, ZIF‐90 with angstrom‐sized pores is a decisive factor in the ionic transport of the MOF/PAA hybrid. Moreover, the fabricated MOF/PAA hybrid exhibited significant ionic rectification over a wide pH range with the maximum ionic rectification ratio of 432 under extreme alkali condition (Figure 5b(ii)). The finite element simulation method was used to investigate the rectification mechanism. It was revealed that the asymmetric geometry and the abrupt surface charge on the hybrid interface influenced the ion current rectification characteristic. Such a low‐cost and facile‐fabrication nanofluidic device exhibiting a high rectification ratio provides a foundational platform to mimic and better understand the ion transport properties, which have potential applications in the fields of biosensing, drug delivery, and energy conversion systems.

### 3.3 Ion Gating

Ionic gating is one of the intrinsic properties of biological ion channels. Ion gating refers to the ability of the ion channels to respond to external stimuli to be opened and closed to adapt to the environment.^[^ ^77^, ^78^ ^]^ Similar to a biological ion channel, when an artificial MOF‐based membrane system is in contact with external stimuli, the “off” and “on” states of ion conduction can be tuned.

**Figure 6:**
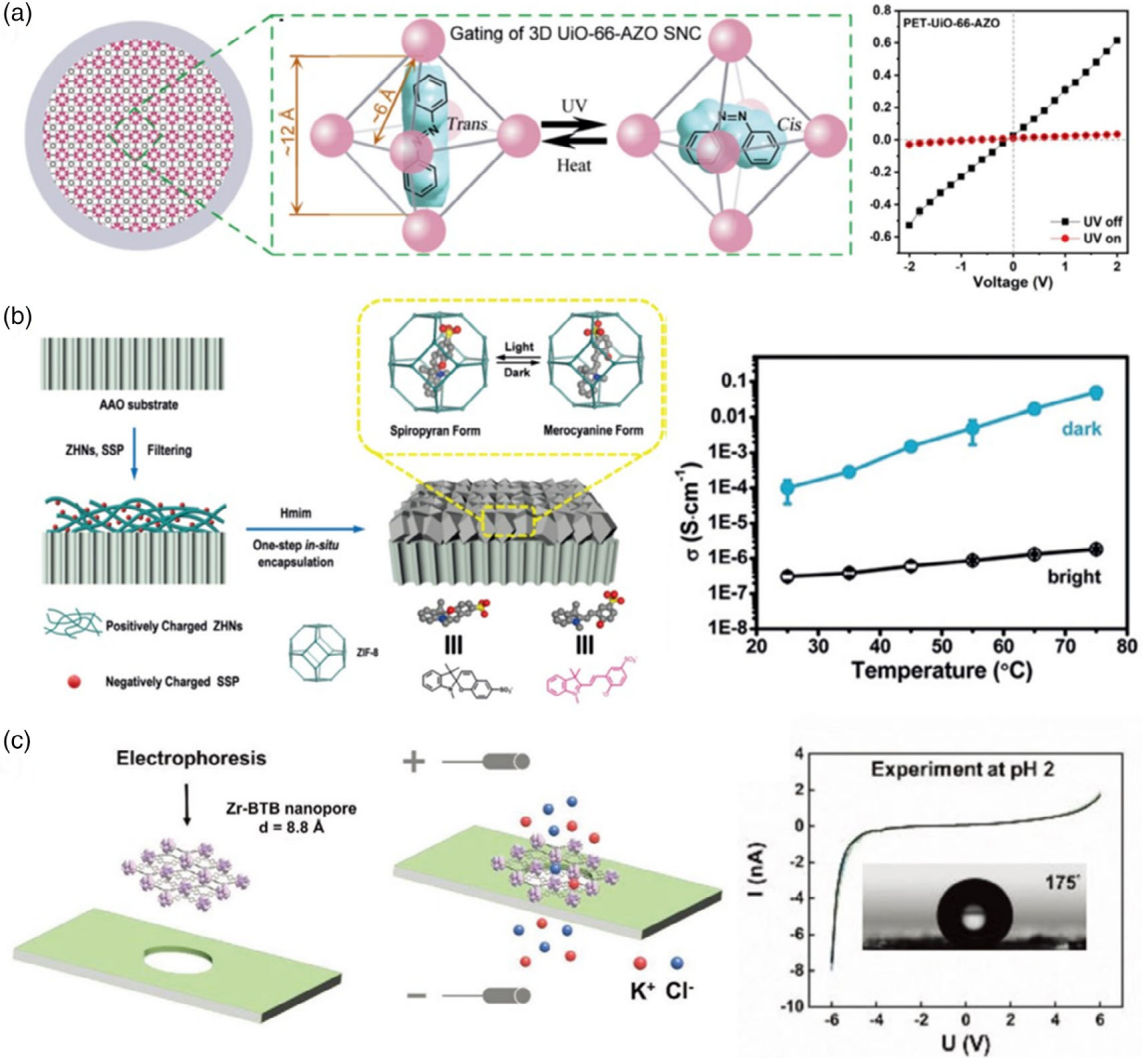
a) Schematic illustration of the reversible conformational switching of the guest AZO molecule inside the 3D MOF subnanochannel, and the *I–V* curves of PET–UiO‐66–AZO channels in the presence and absence of UV irradiation. b) Illustration of the fabrication process of SSP@ZIF‐8 membrane on AAO substrates, and the proton conductivity exhibited by the SSP@ZIF‐8 membrane under visible light and in the dark. c) Schematic illustration of the fabrication steps for the 2D ultrathin MOF nanosheet, and the *I–V* curves of Zr‐based MOF nanopores at pH 2 and pH 8. (a) Reproduced with permission.^[^ ^79^ ^]^ Copyright 2020, Wiley‐VCH. (b) Reproduced with permission.^[^ ^80^ ^]^ Copyright 2020, Wiley‐VCH. (c) Reproduced with permission.^[^ ^82^ ^]^ Copyright 2020, Wiley‐VCH.

Most recently, inspired by nature, Qian et al. fabricated a photoresponsive MOF subnanochannel by integrating azobenzene (AZO) molecules into 3D zirconium‐based UiO‐66 framework confined within single polymer nanochannels.^[^ ^79^ ^]^ Under UV and heat stimuli, the guest AZO molecule inside the Zr‐based UiO‐66 cavity can transform between the *trans*‐AZO and *cis*‐AZO forms, contributing to the ion flux through the channel between open and closed states (**Figure** **6**a). Furthermore, the reversibility and stability of the MOF's subnanochannel has been proved by switching the channel between its open and closed states multiple times. This work provides a platform to achieve high gating performance with potential for application in drug delivery, energy conversion systems, and biosensing.

Most recently, Liang et al. developed light‐switchable proton‐conductive MOF hybrid membranes, which were fabricated by the in situ encapsulation of photoactive molecular valves (sulfonated spiropyran (SSP)) into the cavities of ZIF‐8 (Figure 6b).^[^ ^80^ ^]^ The configuration of SSP can be changed and switched reversibly in response to light, generating different mobile acidic protons. Notably, the SSP@ZIF‐8 membrane exhibited fast response time and outstanding gating properties with an ultrahigh on/off ratio of 2.8 × 10^4^ upon visible light irradiation. This study reports a new strategy for creating emerging smart protonic solids, which have potential applications in many areas such as remote‐controllable chemical sensors and proton‐conducting field‐effect transistors. In addition, Liang and co‐workers used the fabricated SSP@ZIF‐8 membrane for realizing light‐gated lithium ion selective transport, with a high on/off ratio of 23.^[^ ^81^ ^]^

In addition to the light‐gated MOF hybrid membranes, the Gu group developed voltage‐responsive 2D MOF nanosheets, which were synthesized via an electrophoretic method supported by SiN_*x*_ substrates (Figure 6c).^[^ ^82^ ^]^ The fabricated MOF‐based composite nanopores exhibited a voltage‐activated gating phenomenon, with a large gap at 4 V. The high‐conductance state was achieved above 4 V and the low‐conductance state was achieved below 4 V. The emergence of this large gap and the asymmetry of the *I*–*V* curves could be explained by the hydrophobic effect and charge effect influenced by pH. The nonlinear ion transport phenomenon observed in the ultrathin 2D nanosheets offers an opportunity to explore further applications as an ionic diode in solid‐state nanopores.

## 4 Applications

**Figure 7:**
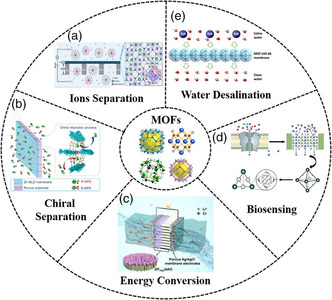
Schematic illustration of various MOFs and their emerging ion transport applications, including a) ion separation, b) chiral separation, c) energy conversion, d) biosensing, as well as e) water desalination. a) Reproduced with permission.^[^ ^92^ ^]^ Copyright 2018, AAAS; b) Reproduced with permission.^[^ ^93^ ^]^ Copyright 2020, Springer Nature; c) Reproduced with permission.^[^ ^85^ ^]^ Copyright 2019, American Chemical Society; d) Reproduced with permission.^[^ ^100^ ^]^ Copyright 2019, Springer Nature; e) Reproduced with permission.^[^ ^104^ ^]^ Copyright 2015, American Chemical Society.

Benefiting from their unique pore structures, high porosity, and outstanding tunability for desired functionalities, artificial MOF‐based membranes exhibit promise in ion transport applications, including biosensing, energy conversion, and ion/molecule separation (**Figure** **7**).

### 4.1 Energy Conversion

**Figure 8:**
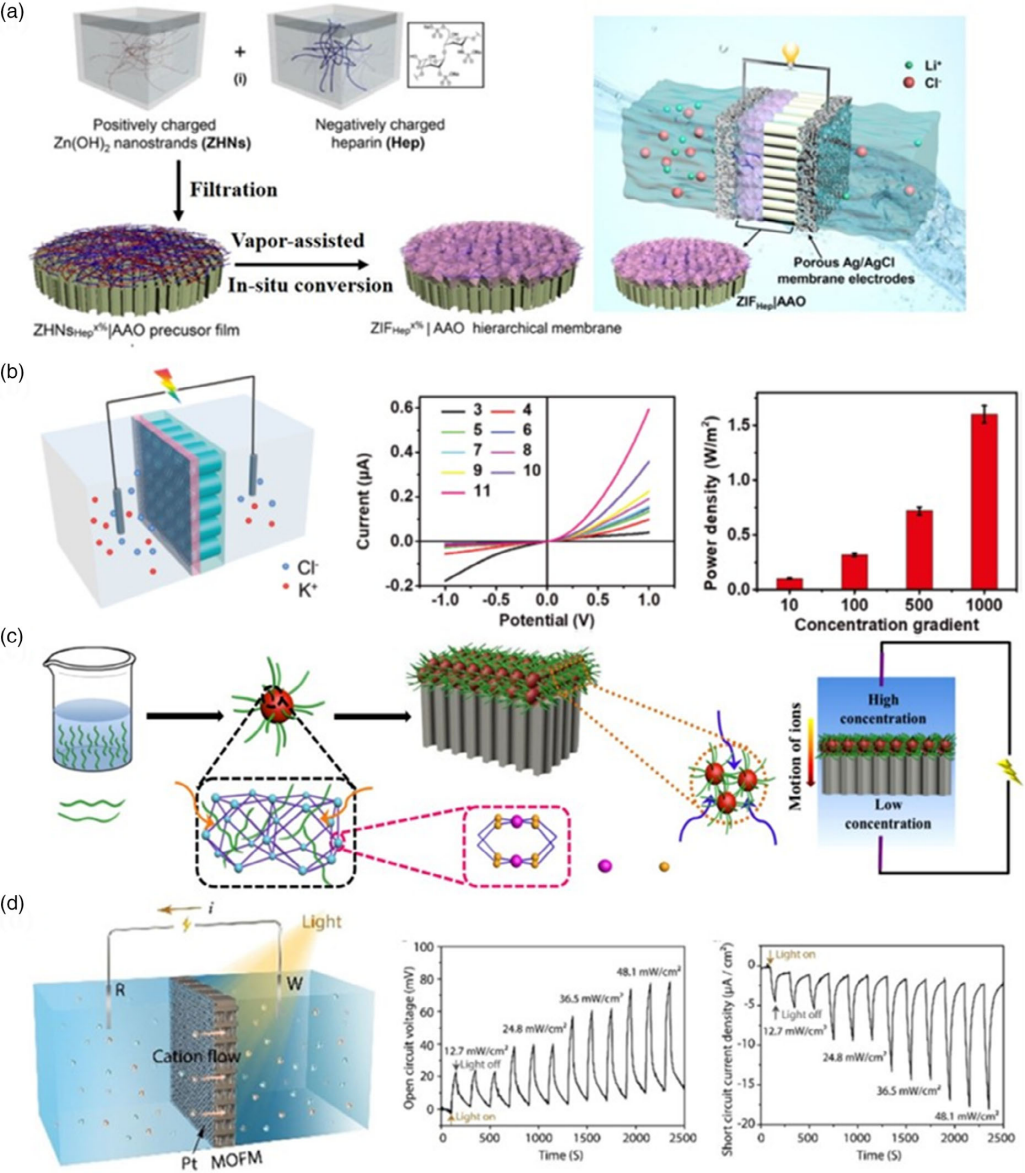
a) Fabrication process of the MOF‐based hybrid membrane and schematic illustration of energy conversion under a concentration gradient. b) Schematic illustration showing the process of energy harvesting under salinity difference, the ionic transport properties of the 2D MOF/PAA hybrid membrane, and the power densities of the hybrid membrane under a series of salinity concentration gradients. c) Schematic diagram of fabricating MOF‐based hybrid membrane by a vapor‐assisted in situ conversion process. And schematic illustration of salinity‐gradient power generator with the MOF–AAO hybrid membrane as the cell separator. d) Sketch map of photodriven active ion transport through an MOF‐based hybrid membrane as well as the open‐circuit voltage and short‐circuit current density generated by active cation transport. (a) Reproduced with permission.^[^ ^85^ ^]^ Copyright 2019, American Chemical Society. (b) Reproduced with permission.^[^ ^86^ ^]^ Copyright 2020, Wiley‐VCH. (c) Reproduced with permission.^[^ ^32^ ^]^ Copyright 2018, Elsevier. (d) Reproduced with permission.^[^ ^90^ ^]^ Copyright 2020, Wiley‐VCH.

MOFs feature well‐defined nanochannels, high porosity, and rich functionalities, which have been developed in energy conversion systems. Salinity gradient power is a promising and clean energy which has become a research hotspot because of the continuing fossil fuel consumption and the increasing energy demands in recent years.^[^ ^83^, ^84^ ^]^ To capture this energy, numerous efforts have been made to design efficient harvesting devices based on MOF‐based smart channels. For example, Guo and co‐workers fabricated an ultrathin, crack‐free zeolite–imidazole framework (ZIF‐8) membrane containing a high density of sulfonated ions via a vapor‐assisted in situ conversion process (**Figure** **8**a).^[^ ^85^ ^]^ The fabricated MOF membrane exhibited excellent selectivity for Li^+^, which was then applied in a salinity‐gradient energy conversion device. At a salinity of 10^5^, such a salinity‐gradient energy conversion device presented a significantly decreased internal resistance (25.6 Ω), and a maximum output power of up to 9.03 μW, which is significantly higher than that of most of the devices reported previously. This work presents an important paradigm for the use of MOF‐based membranes in salinity‐gradient energy conversion and opens new and promising routes to various breakthroughs in the fields of chemistry, biosensing, and nanotechnology.

In addition, Xia et al. reported a facile in situ synthesis method to fabricate hybrid nanochannels constructed by 2D MOFs and PAA, which was used for exploring ion transport and salinity‐gradient energy conversion (Figure 8b).^[^ ^86^ ^]^ The fabricated hybrid membrane exhibited a high ionic rectification ratio over a wide pH range due to the asymmetries in chemical composition and surface charge properties. Moreover, the maximum power output the 2D MOF/PAA hybrid membrane could achieve was as high as 1.6 W m^−2^. This work helps us better understand the ion transport systems and opens up new ways of utilizing channels for energy conversion and water desalination.

Recently, MOF/polymer composite membranes have become promising candidates for the capture of salinity‐gradient power.^[^ ^87^, ^88^ ^]^ MOF/polymer composite membranes are more selective and more efficient ion transporters than pure MOF membranes. Thus, they can generate a higher power density. For example, our group fabricated a hybrid nanochannel membrane by integrating a MOF/polymer composited with AAO (Figure 8c).^[^ ^32^ ^]^ The obtained hybrid nanochannel membranes possess geometrical, chemical, and electrostatic asymmetries, which exhibit diode‐like behavior, rectifying the ion current. We further applied these membranes into a salinity‐gradient‐driven device, which generated an outpower density of up to 2.87 W m^−2^, exhibiting great promise for practical applications. Notably, in a salinity‐gradient‐driven device, the ions can pass through not only the micropores of the MOF, but also the large‐sized gap channels between MOF particles, leading to adequate mass transport. This work reports a general method to fabricate MOF/polymer composite membranes and boosts their applications in energy conversion areas.

In nature, bacteriorhodopsin and proteorhodopsin can convert sunlight into electronic excitation energy by transporting ions from low‐ to high‐concentration regions to generate photovoltage.^[^ ^89^ ^]^ Over the years, great achievements have been obtained with respect to the applications of artificial MOF‐based membrane systems for constructing photoelectric energy conversion devices. Very recently, Mao's group developed a biomimetic photodriven ion pump to realize the energy‐coupled uphill cation transport process by using a porphyrin‐based MOF membrane (Figure 8d).^[^ ^90^ ^]^ The MOF membrane was prepared by in situ generation of metal ions from the AAO template using tetrakis(4‐carboxyphenyl)porphyrin as the linker. Subsequently, the MOF membrane was decorated with Pt particles on one surface to fabricate a Schottky barrier photodiode. Upon light irradiation, the MOF‐based membrane separates electrons and holes, contributing to photovoltage, which increases with increase of the light power density. This work provides an important paradigm for the application of an MOF‐based membrane in photoelectric energy conversion areas and opens up a new and promising route in the fields of ion sieving and artificial photosynthesis.

### 4.2 Ion/Chiral Separation

**Figure 9:**
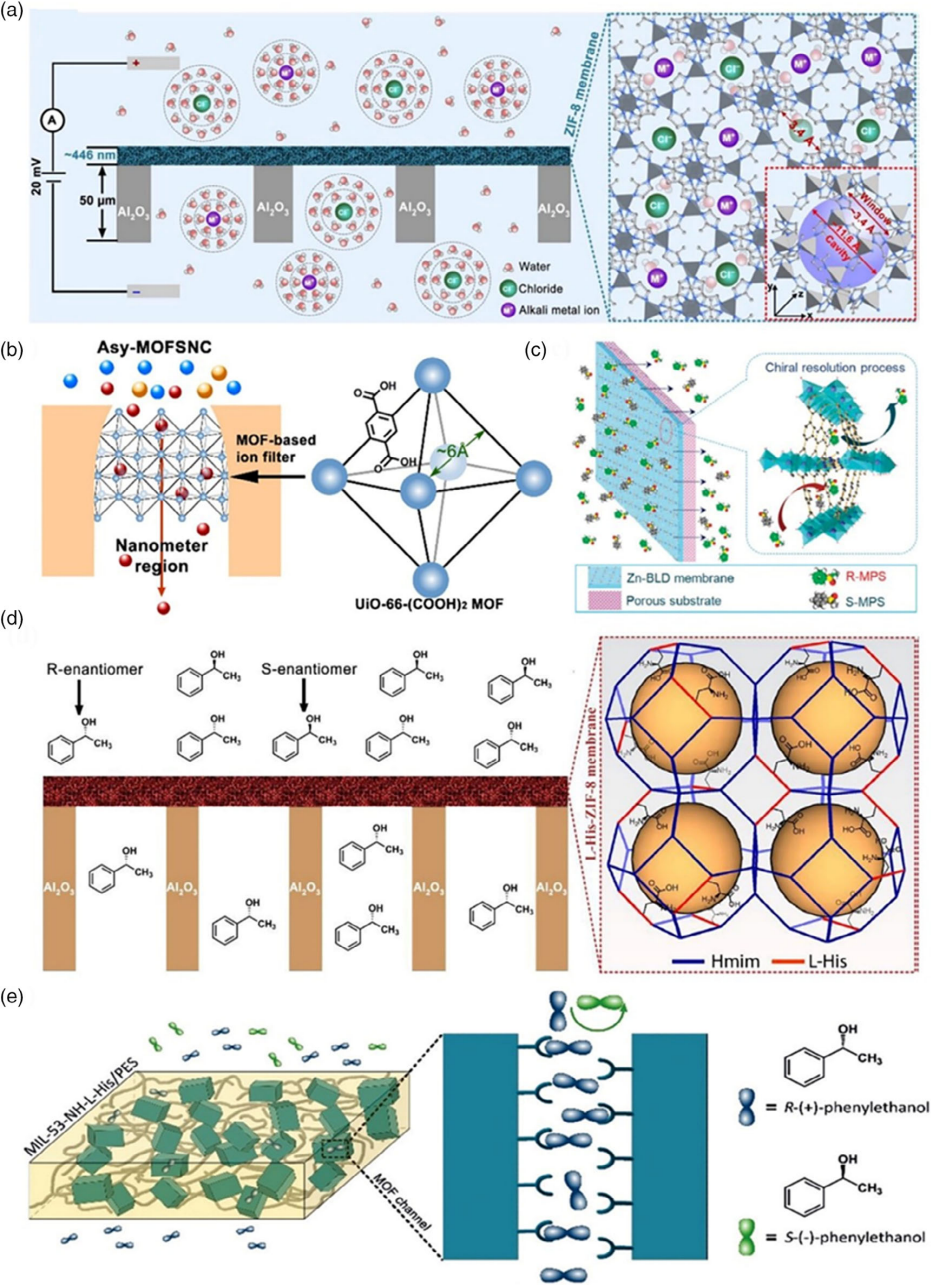
a) Schematic illustration of rapid and selective ion transport through a ZIF‐8/GO/AAO membrane. b) Bioinspired design of an artificial asymmetrical metal organic framework subnanochannel (MOFSNC) fabricated using the facilitated interfacial growth strategy. c) Schematic illustration of a homochiral [Zn_2_(bdc)(l‐lac)(dmf)] (Zn‐BLD) membrane for the enantioselective separation of racemic MPSs. d) Schematic diagram of a homochiral l‐His–ZIF‐8 membrane for separating the *R*‐enantiomer of 1‐phenylethanol from the *S*‐enantiomer. e) Schematic illustration of the selective transport of *R* (+) and *S* (−) 1‐phenylethanol through the MIL‐53–NH–l‐His channel. (a) Reproduced with permission.^[^ ^92^ ^]^ Copyright 2018, AAAS. (b) Reproduced with permission.^[^ ^93^ ^]^ Copyright 2020, Springer Nature. (c) Reproduced with permission.^[^ ^96^ ^]^ Copyright 2012, Royal Society Chemistry. (d) Reproduced with permission.^[^ ^97^ ^]^ Copyright 2018, Wiley‐VCH. (e) Reproduced with permission.^[^ ^98^ ^]^ Copyright 2019, Wiley‐VCH.

Porous membranes exhibiting ultrafast ion permeation and high ion selectivity are urgently required for achieving efficient ion separation, although it is still a huge challenge to efficiently separate monatomic ions of the same valence states and similar sizes using synthetic membranes. Previous studies have demonstrated MOF‐based membranes with diverse subnanometer pore structures and ion selectivities for efficient ion separation applications.^[^ ^91^ ^]^ Recently, Wang's group developed ultrafast selective transport of alkali metal ions in an ultrathin ZIF‐8 membrane fabricated by a nanoporous GO‐assisted interfacial growth method on AAO substrate (**Figure** **9**a).^[^ ^92^ ^]^ This membrane can selectively transport Li^+^ at a very fast rate. The mechanism of ion transport through the ZIF‐8 framework was investigated by molecular dynamics simulation, which demonstrated that ultrafast and selective ion transport in ZIF‐8 is associated with partial dehydration effects. The window size of the ZIF‐8 crystal structure was 3.4 Å, which was larger than the dehydrated ionic diameter and smaller than the hydrated ionic diameter. Thus, the ions get partially dehydrated when transported through the ZIF‐8 membrane and then rehydrated when exiting the channel. The ZIF‐8 membrane exhibited a LiCl/RbCl selectivity of ≈4.6, which is superior to the LiCl/RbCl selectivity of 0.6 in traditional porous membranes. This work reveals the mechanisms of monovalent ion‐selective transport through subnanometer MOF pores and provides a platform for the development of unique MOFs for efficient ion separation.

In addition to separating monovalent ions, it is also important to achieve efficient mono/divalent ion sieving using MOF‐based membranes. Lu et al. fabricated an MOF‐based subnanochannel using a facilitated interfacial growth strategy based on single bullet‐shaped PET membranes (Figure 9b).^[^ ^93^ ^]^ The fabricated asymmetric MOF‐based membrane exhibited a high rectification ratio on account of the asymmetric surface charge distribution and pore size from tip to base. Notably, the asymmetric MOF‐based subnanochannel can rapidly conduct K^+^, Na^+^, and Li^+^ in the subnanometer‐to‐nanometer channel direction, with conductivities up to three orders of magnitude higher than those of Ca^2+^ and Mg^2+^, equivalent to a mono/divalent ion selectivity of 10^3^. Moreover, the theoretical simulation studies revealed that metal ion transport in MOF channels is associated with dehydration–rehydration effects and ion–COOH interactions, both of which could substantially increase the energy barrier for divalent metal ions passing through the MOF‐based subnanochannel, and thus contribute to ultrahigh mono/divalent ion selectivity. This work reports a new route for fabricating diverse multifunctional ion channel membranes for applications such as efficient ion sieving, energy conversion, and power generation.

Apart from ion separation, the MOF‐based membranes have gained considerable attention for chiral separation because the MOFs possess high surface area, uniform pores, as well as highly functionalizable framework structures.^[^ ^94^, ^95^ ^]^ Chiral separation plays an essential role in aspects of medical, biological sciences, food chemistry, and drug delivery. For example, Wang et al. were the first to report a chiral separation membrane, which was fabricated using a combination of homochiral Zn‐BLD and a porous zinc oxide substrate via a reactive seeding technique (Figure 9c).^[^ ^96^ ^]^ The synthesized Zn‐BLD membrane exhibited preferential adsorption ability to (*S*)‐methyl phenyl sulfoxide (*S*‐MPS) over *R*‐MPS, with an enantiomeric excess value of 33%. The chiral environment in the Zn‐BLD membrane was produced by the direct introduction of a chiral ligand. Experimental and simulation results revealed that the separation mechanism of the Zn‐BLD membrane could be attributed to the different interactions of the two enantiomers with the Zn‐BLD inner pores. Such a high enantioselective separation has not been reported to date. This work reports an advanced separation technique for preparing homochiral MOF membranes with high selectivity to solve a major societal demand.

In addition, Chan and co‐workers developed a homochiral ZIF membrane for efficient chiral separation, which was synthesized by integrating l‐histidine into ZIF‐8's framework (Figure 9d).^[^ ^97^ ^]^ Due to the specific interaction between the *S*‐enantiomer and the l‐His–ZIF‐8 membrane, this membrane exhibits excellent chiral selectivity for racemic 1‐phenylethanol, with a high enantiomeric excess value reaching 76%. In addition to the remarkable chiral separation performance, the l‐His–ZIF‐8 membrane exhibits excellent stability, not showing any loss in enantioselectivity after three cycles of separation. The ZIF membrane is the first reported example of a membrane capable of separating chiral compounds. A new method of fabricating homochiral MOFs exhibiting high selectivity was reported. These membranes can be potentially used for practical chiral separation.

Recently, Lu et al. designed a novel homochiral MIL‐53‐based MMM for achieving efficient separation, which was synthesized by the simple combination of homochiral MOF components with polyethersulfone (PES) (Figure 9e).^[^ ^98^ ^]^ The fabricated MIL‐53‐based MMM exhibited excellent enantioselectivity for racemic 1‐phenylethanol with the highest enantiomeric excess value up to 100%. Such high chiral selectivity was ascribed to the MIL‐53–NH–l‐His nanocrystals, which acted as chiral selectors when embedded within the PES matrix. This work paves a new way for exploring the application of homochiral MOF‐based MMMs for efficient chiral separation.

### 4.3 Biosensing

**Figure 10:**
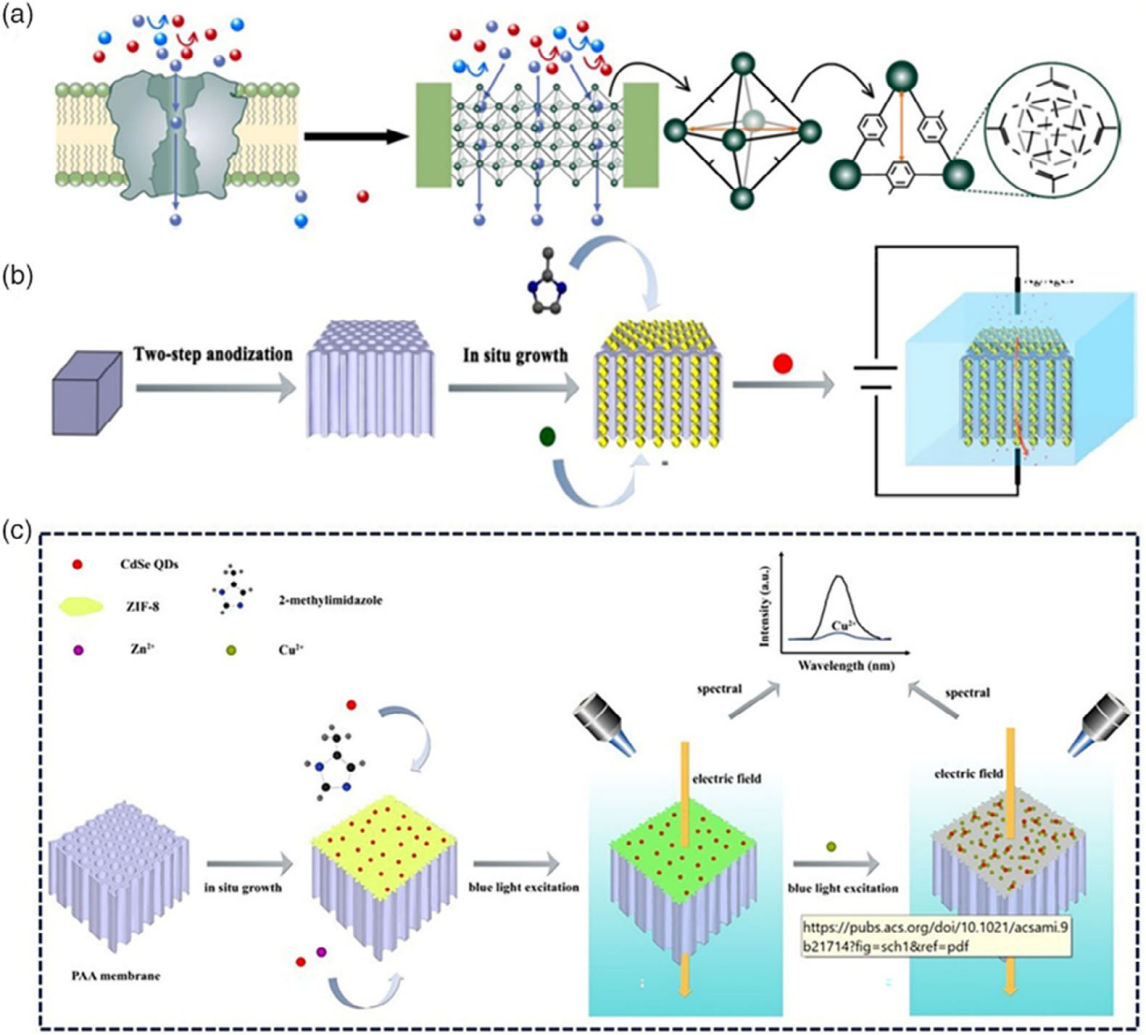
a) Schematic representation showing the biomimetic design of MOF‐based channels for fluoride ion conduction. b) Schematic illustration showing the development of the ZIF‐8/PAA nanochannel composite membrane via an in situ growth method, which was used to detect Pb^2+^. c) Schematic illustration showing the construction of the CdSe@ZIF‐8/PAA membrane and Cu^2+^ detection. (a) Reproduced with permission.^[^ ^100^ ^]^ Copyright 2019, Springer Nature. (b) Reproduced with permission.^[^ ^101^ ^]^ Copyright 2019, American Chemical Society. (c) Reproduced with permission.^[^ ^102^ ^]^ Copyright 2020, American Chemical Society.

To date, numerous efforts have been made toward developing artificial MOF‐based membranes for biochemical sensing, which could lead to the development of next‐generation bioanalytical and diagnostic tools.^[^ ^99^ ^]^ For instance, Li and co‐workers designed highly conductive and selective fluoride ion channels that were constructed by in situ growth of Zr‐based UiO‐66‐derivative MOFs into asymmetric single‐nanochannel PET membranes (**Figure** **10**a).^[^ ^100^ ^]^ The fabricated MOF‐based nanochannels contain specific F^−^ binding sites along the channels, which exhibit ultrahigh F^−^ selectivity and conductivity. Experiment results and molecular dynamic simulations revealed that the remarkably high fluoride selectivity and conductivity in UiO‐66–X channels are attributed to the high concentration of F^−^ ions resulting from the strong interactions between F^−^ ions and the binding sites of the UiO‐66 channels. This work reports an attractive strategy for developing artificial fluoride ion channel membranes. Moreover, MOFs with tailorable angstrom‐sized pores can be potentially used for constructing other types of ion channels and separation membranes.

Most recently, Mo et al. developed ZIF‐8/PAA composite membranes with ultrasmall nanochannels (0.8–1.2 nm) via an in situ growth method (Figure 10b).^[^ ^101^ ^]^ The composite membrane was used as a sensor to detect lead ion (Pb^2+^) by the coordination interaction between Pb^2+^ and nitrogen atoms as ZIF‐8 is a microporous material and has an intense ability to adsorb heavy metals. Furthermore, the detection limit of this sensor reached to 0.03 nm, which can be attributed to the enrichment of nanochannels under an electric field. This work provides guidance for developing an analytical method for sensing heavy metal ions using MOF‐based membranes.

In addition, Gao et al. reported a CdSe@ZIF‐8/PAA membrane for detecting copper ions, which was fabricated using ZIF‐8 and CdSe quantum dot composites on the PAA membrane via an in situ growth approach (Figure 10c).^[^ ^102^ ^]^ The MOF‐based membrane detects copper ions by quenching the fluorescence intensity through the interaction between Cu^2+^ and Se and S atoms. Furthermore, the composite membrane exhibited excellent selectivity for copper ions, with the detection limit reaching 4 fm. This study presents a paradigm for constructing a visualization of an MOF‐based membrane and detection technology for heavy metal ions.

### 4.4 Water Desalination

**Figure 11:**
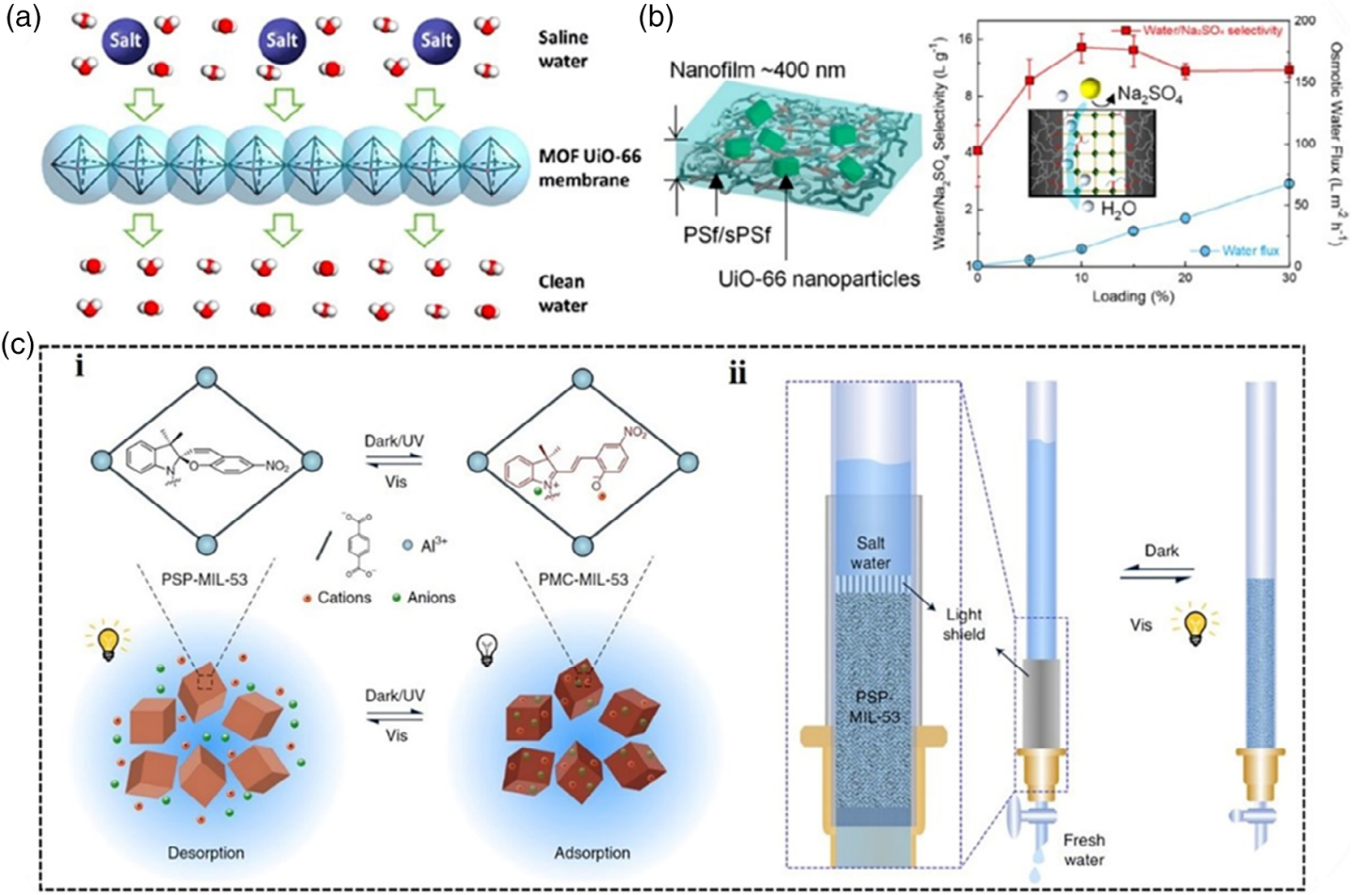
a) Schematic diagram of the UiO‐66‐based framework with the subnanometer channels for water desalination. b) Schematic illustration of the UiO‐66 membrane used for water desalination. c) i. Schematic illustration and mechanism of salt adsorption by PSP–MIL‐53 under dark/UV light and desorption under visible light irradiation. ii. Single‐column setup for desalination in the dark and regeneration under visible light. (a) Reproduced with permission.^[^ ^104^ ^]^ Copyright 2015, American Chemical Society. (b) Reproduced with permission.^[^ ^105^ ^]^ Copyright 2018, American Chemical Society. (c) Reproduced with permission.^[^ ^106^ ^]^ Copyright 2020, Springer Nature.

MOF‐based membranes with well‐defined pores, unique pore structures, and angstrom‐sized windows can act as ion selectivity filters for sieving metal ions, which are promising candidates for the removal of common cations and anions from water to achieve efficient water desalination.^[^ ^103^ ^]^ In 2015, Li's group fabricated a Zr‐MOF‐based membrane on alumina hollow fibers via an in situ solvothermal synthesis method. The membrane exhibited moderate permeance (0.14 L m^−2^ h^−1^ bar^−1^) and good permeability (0.28 L m^−2^ h^−1^ bar^−1^) due to the size exclusion with excellent multivalent ion rejection (**Figure** **11**a).^[^ ^104^ ^]^ The membrane exhibits high separation performance and possesses outstanding water stability. These properties make the fabricated UiO‐66 membrane a promising candidate for water desalination. Recently, Liu et al. developed a self‐standing UiO‐66‐based nanocomposite membrane using a facile solution‐casting and annealing approach, which realized high osmotic water permeability up to 1.41 L m^−2^ h^−1^ bar^−1^, and enhanced water/Na_2_SO_4_ permselectivity of 13.5 L g^−1^ (Figure 11b).^[^ ^105^ ^]^

Particularly, Wang's group reported a poly(spiropyran acrylate) (PSP)‐functionalized MOF as a sunlight‐regenerable ion adsorbent for sustainable desalination.^[^ ^106^ ^]^ Under dark conditions, the zwitterionic isomer can quickly adsorb multiple cations and anions from water within 30 min, and it isomerized to SP and quickly released these adsorbed salts within 4 min under sunlight illumination, with high ion adsorption loadings of up to 2.88 mmol g^−1^ of NaCl (Figure 11c(i)). Moreover, the single‐column desalination experiments revealed that the PSP–MOF works efficiently for water desalination (Figure 11c(ii)). Notably, the PSP–MOF exhibited excellent stability and cycling performance. This study offers valuable insight into the design of stimuli‐responsive materials that can be potentially used for sustainable water desalination.

## 5 Conclusion

Learning from nature is the eternal theme for developing novel intelligent materials and new smart systems. In this article, we reviewed the recent advances in the field of the development of artificial MOF‐based membranes that can be potentially used for ion transport studies. Moreover, a strategy for designing and constructing artificial MOF‐based membranes, including free‐standing MOF membranes, MOF/polymer MMMs, and MOF membrane coating on substrates, is proposed. We also reviewed the excellent ionic transport characteristics based on MOF membranes, including ionic selectivity, ionic rectification, and ionic gating. These unique properties will open up emerging ion transport applications of MOF‐based membranes such as energy conversion, ion/molecule separation, biochemical sensing, and water desalination.

Despite these remarkable achievements in recent years, artificial MOF‐based membranes still face challenges in practical applications, as investigations of material preparation, transport mechanisms, and device integration are still in their initial stage. For example, the mechanism of ion transport across the MOF‐based membrane needs to be further investigated in detail. Further research should also be conducted to figure out the relationship between the MOF pore structures, the functional groups, and the ion transport performance. Thus, computational simulation methods need to be developed to investigate the ion transport mechanisms through MOF membranes. Moreover, the structure of MOFs can be modified with different functional groups, such as (NH_2_, —COOH, —OH, and —SO_4_). MOF‐based hybrid materials with a polymer or porous substrate, which could effectively improve the ion selectivity of the MOF‐based membranes. In addition, MOF membranes with high porosity, well‐defined pore sizes, and unique pore structures of MOF crystals can be fabricated with existing methods, although the mechanical properties of these membranes are still not suitable for the harsh work environment in practical applications due to the intrinsic frangibility of MOF crystals. The intricate fabrication processes can be realized in a laboratory, although it is experimentally challenging to complete large‐scale manufacturing in a factory. Thus, it is essential and urgent to fabricate low‐cost, stable, environment‐friendly, and high‐quality MOF‐based membranes.

As MOFs possess angstrom‐sized pores, the ion transportation phenomenon is different from the pores with nano‐sized, quantum‐confined and ionic coulomb blockade effect can be observed, which can offer excellent platform to realize functions analogous to the biological membranes. More effort needs to be made in the future to enhance the selectivity, the rectification property, as well as the gating ability of MOF‐based membranes to realize more intelligent artificial nanochannels. Furthermore, research will continue to find a more effective approach to minimize the defects of membranes for large‐scale manufacturing. Collective research efforts from multidisciplinary areas including chemistry, physics, and material science are needed to promote the commercialization of advanced MOF membranes.

## Conflict of Interest

The authors declare no conflict of interest.

## References

[1] E. Barry , S. P. McBride , H. M. Jaeger , X. M. Lin , Nat. Commun. 2014, 5, 5847. 25517763 10.1038/ncomms6847

[2] Z. W. Yao , B. F. Qiao , M. O. de le Cruz , Phys. Rev. E 2013, 88, 062712. 10.1103/PhysRevE.88.062712 24483491

[3] J. H. Morais-Cabral , Y. F. Zhou , R. Mackinnon , Nature 2001, 414, 37. 11689935 10.1038/35102000

[4] J. Xu , D. A. Lavan , Nat. Nanotechnol. 2008, 3, 666. 18989332 10.1038/nnano.2008.274 PMC2767210

[5] Y. L. Xu , X. Sui , S. Guan , J. Zhai , L. C. Gao , Adv. Mater., 2015, 27, 1851. 25649041 10.1002/adma.201405564

[6] X. Sui , Z. Zhang , C. Li , L. C. Gao , Y. Zhao , L. J. Yang , L. P. Wen , L. Jiang , ACS. Appl. Mater. Interfaces 2019, 11, 23815. 30035526 10.1021/acsami.8b02578

[7] R. Li , X. Sui , C. Li , J. Q. Jiang , J. Zhai , L. C. Gao , ACS Appl. Mater. Interfaces 2018, 10, 3241. 29303249 10.1021/acsami.7b14505

[8] L. L. Fu , J. Q. Jiang , B. X. Lu , Y. L. Xu , J. Zhai , ChemNanoMat 2019, 5, 1182.

[9] Z. Y. Meng , Y. Chen , X. L. Li , Y. L. Xu , J. Zhai , ACS Appl. Mater. Interfaces 2015, 7, 7709. 25806828 10.1021/acsami.5b00647

[10] Q. Q. Zhang , Q. R. Liu , J. X. Kang , Q. J. Huang , Z. Y. Liu , X. G. Diao , J. Zhai , Adv. Sci. 2018, 5, 1800163. 10.1002/advs.201800163 PMC6145424 30250783

[11] M. H. Zhang , X. Hou , J. T. Wang , Y. Tian , X. Fan , J. Zhai , L. Jiang , Adv. Mater. 2012, 24, 2424. 22488964 10.1002/adma.201104536

[12] K. Xiao , L. Chen , R. T. Chen , T. Heil , S. D. C. Lemus , F. T. Fan , L. P. Wen , L. Jiang , M. Antonietti , Nat. Commun. 2019, 10, 74. 30622279 10.1038/s41467-018-08029-5 PMC6325115

[13] J. Gao , W. Guo , D. Feng , H. T. Wang , D. Y. Zhao , L. Jiang , J. Am. Chem. Soc, 2014, 136, 12265. 25137214 10.1021/ja503692z

[14] Q. Q. Zhang , T. L. Xiao , N. N. Yan , Z. Y. Liu , J. Zhai , X. G. Diao , Nano Energy, 2016, 28, 188.

[15] K. Xiao , K. Wu , L. Chen , X. Y. Kong , Y. Q. Zhang , L. P. Wen , L. Jiang , Angew. Chem., Int. Ed. 2018, 130, 157.

[16] Shang, X. M. , Xie, G. H. , Kong, X. Y. , Zhang, Z. , Zhang, Y. Q. , Tian, W. , Wen, L. P. , Jiang, L. , Adv. Mater. 2017, 29, 1603884. 10.1002/adma.201603884 27786377

[17] W. Guan , R. Fan , M. A. Reed , Nat. Commun. 2011, 2, 506. 22009038 10.1038/ncomms1514

[18] T. Kitao , Y. Zhang , S. Kitagawa , B. Wang , T. Uemura , Chem. Soc. Rev. 2017, 46, 3108. 28368064 10.1039/c7cs00041c

[19] Z. Zhang , L. Wen , L. Jiang , Chem. Soc. Rev. 2018, 47, 322. 29300401 10.1039/c7cs00688h

[20] S. Horike , D. Umeyama , S. Kitagawa , Acc. Chem. Res. 2013, 46, 2376. 23730917 10.1021/ar300291s

[21] J. Hou , H. T. Wang , H. C. Zhang , Ind. Eng. Chem. Res. 2020, 59, 12907.

[22] H. Kazemzadeh , J. Karimi-Sabet , J. T. Darian , A. Adhami , Sep. Purif. Technol. 2020, 251, 117298.

[23] Y. B. He , Y. Qiao , Z. Chang , H. S. Zhou , Energy Environ. Sci. 2019, 12, 2327.

[24] T. J. Qiu , Z. B. Liang , W. H. Guo , H. Tabassum , S. Gao , R. Q. Zou , ACS Energy Lett. 2020, 5, 520.

[25] J. Hou , H. C. Zhang , G. P. Simon , H. T. Wang , Adv. Mater. 2019, 32, 1902009. 10.1002/adma.201902009 31273835

[26] J. Li , H. Wang , X. Z. Yuan , J. Zhang , J. W. Chew , Coord. Chem. Rev. 2020, 404, 213116.

[27] X. J. Ma , Y. T. Chai , P. Li , B. Wang , Acc. Chem. Res. 2019, 52, 1461. 31074608 10.1021/acs.accounts.9b00113

[28] J. F. Yao , H. T. Wang , Chem. Soc. Rev. 2014, 43, 4470. 24668302 10.1039/c3cs60480b

[29] L. Gao , K. Y. Chan , C. Y. V. Li , L. X. Xie , J. F. Olorunyomi , Nano Lett. 2019, 19, 4990. 31322897 10.1021/acs.nanolett.9b01211

[30] Y. Guo , X. S. Peng , Sci. China Mater. 2019, 62, 25.

[31] G. L. Yu , X. Q. Zou , L. Sun , B. S. Liu , Z. Y. Wang , P. Zhang , G. S. Zhu , Adv. Mater. 2019, 31, 1806853. 10.1002/adma.201806853 30803076

[32] R. Li , J. Q. Jiang , Q. Liu , Z. Q. Xie , J. Zhai , Nano Energy 2018, 53, 643.

[33] M. Bosch , S. Yuan , W. Rutledge , H. C. Zhou , Acc. Chem. Res. 2017, 50, 857. 28350434 10.1021/acs.accounts.6b00457

[34] H. X. Ang , L. Hong , ACS Appl. Mater. Interfaces 2017, 9, 28079. 28752999 10.1021/acsami.7b08383

[35] H. Wang , S. Zhao , Y. Liu , R. X. Yao , X. Q. Wang , Y. H. Cao , D. Ma , X. Feng , B. Wang , Nat. Commun., 2019, 10, 4204. 31527592 10.1038/s41467-019-12114-8 PMC6746862

[36] D. Bradshaw , A. Garai , J. Huo , Chem. Soc. Rev. 2012, 41, 2344. 22182916 10.1039/c1cs15276a

[37] O. Shekhah , J. Liu , R. A. Fischer , C. Woll , Chem. Soc. Rev. 2011, 40, 1081. 21225034 10.1039/c0cs00147c

[38] X. Y. Li , H. C. Zhang , J. Hou , R. W. Ou , Y. L. Zhu , C. Zhao , T. Y. Qian , C. D. Easton , C. Selomulya , M. R. Hill , H. T. Wang , J. Am. Chem. Soc. 2020, 142, 9827. 32364714 10.1021/jacs.0c03554

[39] Y. F. Shu , J. N. Hao , D. C. Niu , Y. S. Li , J. Mater. Chem. C 2020, 8, 8635.

[40] X. Y. Chen , D. Y. Chen , N. J. Li , Q. F. Xu , H. Li , J. H. He , J. M. Lu , ACS Appl. Mater. Interfaces 2020, 12, 39227. 32805808 10.1021/acsami.0c10290

[41] G. R. Lee , H. Ohtsu , J. Koo , Y. Yahiyama , M. J. Park , D. Inoue , D. Hashizumec , M. Kawano , Chem. Commun. 2016, 52, 3962. 10.1039/c5cc10136k 26882279

[42] A. Fateeva , P. A. Chater , C. P. Ireland , A. A. Tahir , Y. Z. Khimyak , P. V. Wiper , J. R. Darwent , M. J. Rosseinsky , Angew. Chem., Int. Ed. 2012, 51, 7440. 10.1002/anie.201202471 22696508

[43] M. P. Jian , R. S. Qiu , Y. Xia , J. Lu , Y. Chen , Q. F. Gu , R. P. Liu , C. Z. Hu , H. T. Wang , X. W. Zhang , Sci. Adv. 2020, 6, eaay3998. 32548253 10.1126/sciadv.aay3998 PMC7274808

[44] J. Hou , H. C. Zhang , G. P. Simon , H. T. Wang , Adv. Mater. 2020, 32, 1902009. 10.1002/adma.201902009 31273835

[45] R. Ou , H. Zhang , J. Wei , S. Kim , L. Wan , N. S. Nguyen , Y. Hu , X. Zhang , G. P. Simon , H. T. Wang , Adv. Mater. 2018, 30, 1802767. 10.1002/adma.201802767 29989209

[46] R. Ameloot , F. Vermoortele , W. Vanhove , M. B. J. Roeffaers , B. F. Sels , D. E. Se Vos , Nat. Chem., 2011, 3, 382. 21505497 10.1038/nchem.1026

[47] X. J. Bai , D. Chen , L. L. Li , L. Shao , W. X. He , H. Chen , Y. N. Li , X. M. Zhang , L. Y. Zhang , T. Q. Wang , Y. Fu , W. Qi , ACS Appl. Mater. Interfaces 2018, 10, 25960. 30051709 10.1021/acsami.8b09812

[48] J. Y. Su , W. F. Wu , Z. J. Li , W. B. Li , Dalton Trans., 2019, 48, 11196. 31298241 10.1039/c9dt02359c

[49] G. D. Wu , J. H. Huang , Y. Zhang , J. He , G. Xu , J. Am. Chem. Soc. 2017, 139, 1360. 27794592 10.1021/jacs.6b08511

[50] D. Kim , M. Y. Jeon , B. L. Stottrup , M. Tsapatsis , Angew. Chem., Int. Ed. 2018, 57, 480. 10.1002/anie.201708835 29194920

[51] S. Bai , X. Liu , K. Zhu , S. Wu , H. Zhou , Nat. Energy 2016, 1, 16094.

[52] Y. He , Z. Chang , S. Wu , Y. Qiao , S. Bai , K. Jiang , P. He , H. Zhou , Adv. Energy Mater. 2018, 8, 1802130.

[53] W. Liu , Y. Mi , Z. Weng , Y. Zhong , Z. Wu , H. Wang , Chem. Sci. 2017, 8, 4285. 28626566 10.1039/c7sc00668c PMC5468994

[54] J. Liu , C. Woll , Chem. Soc. Rev. 2017, 46, 5730. 28682401 10.1039/c7cs00315c

[55] Y. Chen , S. Li , X. Pei , J. Zhou , X. Feng , S. Zhang , Y. Cheng , H. Li , R. Han , B. Wang , Angew. Chem., Int. Ed. 2016, 55, 3419. 10.1002/anie.201511063 26847472

[56] Y. Zang , F. Pei , J. Huang , Z. Fu , G. Xu , X. Fang , Adv. Energy Mater., 2018, 8, 1802052.

[57] A. Centrone , Y. Yang , S. Speakman , L. Bromberg , G. C. Rutledge , T. A. Hatton , J. Am. Chem. Soc. 2010, 132, 15687. 20945899 10.1021/ja106381x

[58] H. Wang , S. Chen , L. Li , S. Jiang , Langmuir 2005, 21, 2633. 15779923 10.1021/la046810w

[59] L. Shi , T. Wang , H. Zhang , K. Chang , J. Ye , Adv. Funct. Mater. 2015, 25, 5360.

[60] J. Liu , W. Zhou , J. Liu , I. Howard , G. Kilibarda , S. Schlabach , D. Coupry , M. Addicoat , S. Yoneda , Y. Tsutsui , Angew. Chem., Int. Ed. 2015, 54, 7441. 10.1002/anie.201501862 25960115

[61] I. Stassen , M. Styles , G. Grenci , H. V. Gorp , W. Vanderlinden , S. D. Feyter , P. Falcaro , D. D. Vos , P. Vereeckenand , R. Ameloot , Nat. Mater. 2016, 15, 304. 26657328 10.1038/nmat4509

[62] Z. Y. Jiang , H. L. Liu , S. A. Ahmed , S. Hanif , S. B. Ren , J. J. Xu , H. Y. Chen , X. H. Xia , K. Wang , Angew. Chem., Int. Ed., 2017, 56, 4767. 10.1002/anie.201701279 28345204

[63] Y. X. Hu , J. Wei , Y. Liang , H. C. Zhang , X. W. Zhang , W. Shen , H. T. Wang , Angew. Chem., Int. Ed. 2016, 55, 2048. 10.1002/anie.201509213 26710246

[64] S. Zhou , Y. Y. Wei , L. B. Li , Y. F. Duan , Q. Q. Hou , L. L. Zhang , L. X. Ding , J. Xue , H. H. Wang , J. Caro , Sci. Adv. 2018, 4, eaau1393. 30410983 10.1126/sciadv.aau1393 PMC6218190

[65] S. C. Hess , R. N. Grass , W. J. Stark , Chem. Mater. 2016, 28, 7638.

[66] Y. Liu , G. P. Liu , C. Zhang , W. L. Qiu , S. L. Yi , V. Chernikova , Z. J. Chen , Y. Belmabkhout , O. Shekhah , M. Eddaoudi , Adv. Sci. 2018, 5, 1800982. 10.1002/advs.201800982 PMC6145261 30250815

[67] G. L. Yu , X. Q. Zou , L. Sun , B. S. Liu , Z. Y. Wang , P. P. Zhang , G. S. Zhu , Adv. Mater. 2019, 31, 1806853. 10.1002/adma.201806853 30803076

[68] Y. Guo , Y. L. Ying , Y. Y. Mao , X. S. Peng , B. L. Chen , Angew. Chem., Int. Ed. 2016, 128, 1.

[69] L. Y. Wang , M. Q. Fang , J. Liu , J. He , J. D. Li , J. D. Lei , ACS Appl. Mater. Interfaces 2015, 7, 24082. 26485228 10.1021/acsami.5b07128

[70] Z. Zhang , X. D. Huang , Y. C. Qian , W. P. Chen , L. P. Wen , L. Jiang , Adv. Mater. 2020, 32, 1904351. 10.1002/adma.201904351 31793736

[71] X. B. Zhu , Y. H. Zhou , J. R. Hao , B. Bao , X. J. Bian , X. Y. Jiang , J. H. Pang , H. B. Zhang , Z. H. Jiang , L. Jiang , ACS Nano 2017, 11, 10816. 29039923 10.1021/acsnano.7b03576

[72] L. Lin , J. Yan , J. H. Li , Anal. Chem. 2014, 86, 10546. 25268828 10.1021/ac501983a

[73] X. Y. Li , H. C. Zhang , Y. Hao , J. Xia , Y. B. Zhu , H. Wu , J. Hou , J. Lu , R. W. Ou , C. D. Easton , C. Selomulya , M. R. Hill , L. Jiang , H. T. Wang , Adv. Mater. 2020, 32, 2001777. 10.1002/adma.202001777 32390263

[74] W. Guo , Y. Tian , Acc. Chem. Res. 2013, 46, 2834. 23713693 10.1021/ar400024p

[75] R. Li , B. X. Lu , Z. Q. Xie , J. Zhai , Small 2019, 15, 1904866. 10.1002/smll.201904866 31778019

[76] F. Liu , Y. C. Guo , W. Wang , Y. M. Chen , C. Wang , Nanoscale 2020, 12, 11899. 32236224 10.1039/d0nr01054e

[77] A. Seifert , K. Gopfrich , J. R. Burns , N. Fertig , U. F. Keyser , S. Howorka , ACS Nano 2015, 9, 1117. 25338165 10.1021/nn5039433 PMC4508203

[78] X. Hou , Adv. Mater. 2016, 28, 7049. 27296766 10.1002/adma.201600797

[79] T. Y. Qian , H. C. Zhang , X. Y. Li , J. Hou , C. Zhao , Q. F. Gu , H. T. Wang , Angew. Chem., Int. Ed. 2020, 59, 2. 10.1002/anie.202004657 32343468

[80] H. Q. Liang , Y. Guo , Y. S. Shi , X. S. Peng , B. Liang , B. L. Chen , Angew. Chem., Int. Ed. 2020, 59, 7732. 10.1002/anie.202002389 32090427

[81] H. Q. Liang , Y. Guo , X. S. Peng , B. L. Chen , J. Mater. Chem. A 2020, 8, 11399.

[82] Q. Zhang , P. S. Cao , Y. Cheng , S. Yang , Y. D. Yin , T. Y. Lv , Z. Y. Gu , Adv. Funct. Mater. 2020, 30, 2004854.

[83] X. B. Zhu , J. R. Hao , B. Bao , Y. H. Zhou , H. B. Zhang , J. H. Pang , Z. H. Jiang , L. Jiang , Sci. Adv. 2018, 4, eaau 1665. 10.1126/sciadv.aau1665 PMC6203222 30397649

[84] C. C. Yu , X. B. Zhu , C. Y. Wang , Y. H. Zhou , X. T. Jia , L. Jiang , X. Liu , G. G. Wallace , Nano Energy 2018, 53, 475.

[85] Y. Guo , H. B. Huang , Z. Y. Li , X. B. Wang , P. P. Li , Z. Deng , X. S. Peng , ACS Appl. Mater. Interfaces 2019, 11, 35496. 31469536 10.1021/acsami.9b13617

[86] C. Wang , F. Liu , Z. Tan , Y. M Chen , W. C. Hu , X. H. Xia , Adv. Funct. Mater. 2020, 30, 1908804.

[87] G. R. Lee , H. Ohtsu , J. Koo , Y. Yakiyama , M. J. Park , D. Inoue , D. Hashizumec , M. Kawano , Chem. Commun. 2016, 52, 3962. 10.1039/c5cc10136k 26882279

[88] K. Fujie , K. Otsubo , R. Ikeda , T. Yamadab , H. Kitagawa , Chem. Sci. 2015, 6, 4306. 29218200 10.1039/c5sc01398d PMC5707510

[89] Z. Zhang , X. Y. Kong , G. H. Xie , P. Li , K. Xiao , L. P. Wen , L. Jiang , Sci. Adv. 2016, 2, e1600689. 27774511 10.1126/sciadv.1600689 PMC5072182

[90] Y. N. Jiang , W. J. Ma , Y. J. Qiao , Y. F. Xue , J. H. Lu , J. Gao , N. N. Liu . F. Wu , P. Yu , L. Jiang , L. Q. Mao , Angew. Chem., Int. Ed. 2020, 59, 1. 10.1002/anie.202005084 32343466

[91] C. Y. Zhang , Y. X. Mu , W. Zhang , S. Zhao , Y. X. Wang , J. Membr. Sci. 2020, 596, 117724.

[92] H. C. Zhang , J. Hou , Y. X. Hu , P. Y. Wang , R. W. Ou , L. Jiang , J. Z. Liu , B. D. Freeman , A. J. Hill , H. T. Wang , Sci. Adv. 2018, 4, eaaq0066. 29487910 10.1126/sciadv.aaq0066 PMC5817922

[93] J. Lu , H. C. Zhang , J. Hou , X. Y. Li , X. Y. Hu , Y. X. Hu , C. D. Easton , Q. Y. Li , C. H. Sun , A. W. Thornton , M. R. Hill , X. W. Zhang , G. P. Jiang , J. Z. Liu , A. J. Hill , B. D. Freeman , L. Jiang , H. T. Wang , Nat. Mater. 2020, 19, 767. 32152561 10.1038/s41563-020-0634-7

[94] K. Huang , X. L. Dong , R. F. Ren , W. Q. Jin , AIChE J. 2013, 59, 4364.

[95] Z. X. Kang , M. Xue , L. Fan , J. Y. Ding , L. J. Guo , L. X. Gao , S. L. Qiu , Chem. Commun. 2013, 49, 10569. 10.1039/c3cc42376j 23792620

[96] W. J. Wang , X. L. Dong , J. P. Nan , W. Q. Jin , Z. Q. Hu , Y. F. Chen , J. W. Jiang , Chem. Commun. 2012, 48, 7022. 10.1039/c2cc32595k 22575898

[97] J. Y. Chan , H. C. Zhang , Y. D. Nolvachai , Y. X. Hu , H. J. Zhu , M. Forsyth , Q. F. Gu , D. E. Hoke , X. W. Zhang , P. Marriot , H. T. Wang , Angew. Chem., Int. Ed. 2018, 21, 17130. 10.1002/anie.201810925 30370963

[98] Y. Lu , H. C. Zhang , J. Y. Chan , R. W. Ou , H. J. Zhu , M. Forsyth , E. M. Marijanovic , C. M. Doherty , P. J. Marriott , M. M. Banaszak , H. T. Wang , Angew. Chem., Int. Ed. 2019, 58, 16928. 10.1002/anie.201910408 31535784

[99] M. Liu , J. S. Mou , X. H. Xu , F. F. Zhang , J. F. Xia , Z. H. Wang , Talanta 2020, 220, 121374. 32928400 10.1016/j.talanta.2020.121374

[100] X. Y. Li , H. C. Zhang , P. Y. Wang , J. Hou , J. Lu , C. D. Easton , X. W. Zhang , M. R. Hill , A. W. Thornton , J. Z. Liu , B. D. Freeman , A. J. Hill , L. Jiang , H. T. Wang , Nat. Commun. 2019, 10, 2490. 31186413 10.1038/s41467-019-10420-9 PMC6560108

[101] R. J. Mo , L. He , C. X. Zhou , Z. J. Qian , P. Z. Hong , S. L. Sun , Z. Wang , Y. Wang , C. Y. Li , Anal. Chem. 2019, 91, 8184. 31140271 10.1021/acs.analchem.9b00638

[102] H. L. Gao , R. K. Sun , L. He , Z. J. Qian , C. X. Zhou , P. Z. Hong , S. L. Sun , R. J. Mo , C. Y. Li , ACS Appl. Mater. Interfaces 2020, 12, 4849. 31904212 10.1021/acsami.9b21714

[103] X. T. Li , N. X. Wang , X. M. Bao , Q. Li , J. Li , Y. B. Xie , S. L. Ji , J. Y. Yuan , Q. F. An , J. Mater. Chem. A 2020, 8, 17212.

[104] X. L. Liu , N. K. Demir , Z. T. Wu , K. Li , J. Am. Chem. Soc. 2015, 137, 6999. 26023819 10.1021/jacs.5b02276

[105] T. Y. Liu , H. G. Yuan , Y. Liu , D. Gen , Y. C. Su , X. L. Wang , ACS Nano 2018, 12, 9253. 30153418 10.1021/acsnano.8b03994

[106] R. W. Ou , H. C. Zhang , V. X. Truong , L. Zhang , H. M. Hegab , H. Li , J. Hou , X. W. Zhang , A. Deletic , L. Jiang , G. P. Simon , H. T. Wang , Nat. Sustain. 2020, 437, 3738.

